# Supplementary figures and images for: The role of seasonal malaria chemoprevention in the effect of azithromycin on child mortality: A secondary analysis of the CHAT cluster randomized clinical trial
Source: PLOS Glob Public Health. 2025 Sep 29;5(9):e0004653. doi: 10.1371/journal.pgph.0004653 (PMC12478956; doi:10.1371/journal.pgph.0004653)

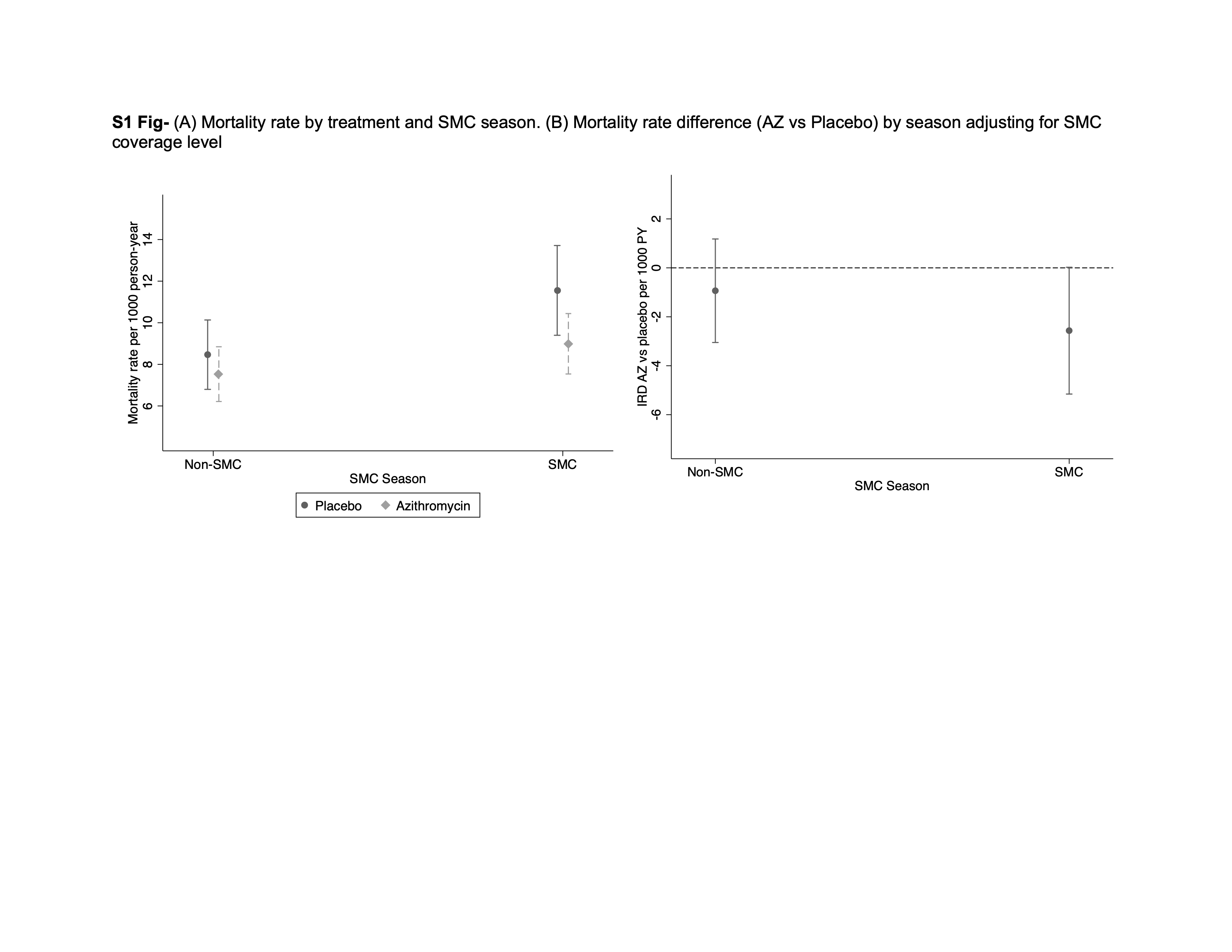

Supplement: S1 Fig — (TIFF) [file pgph.0004653.s002.tiff]

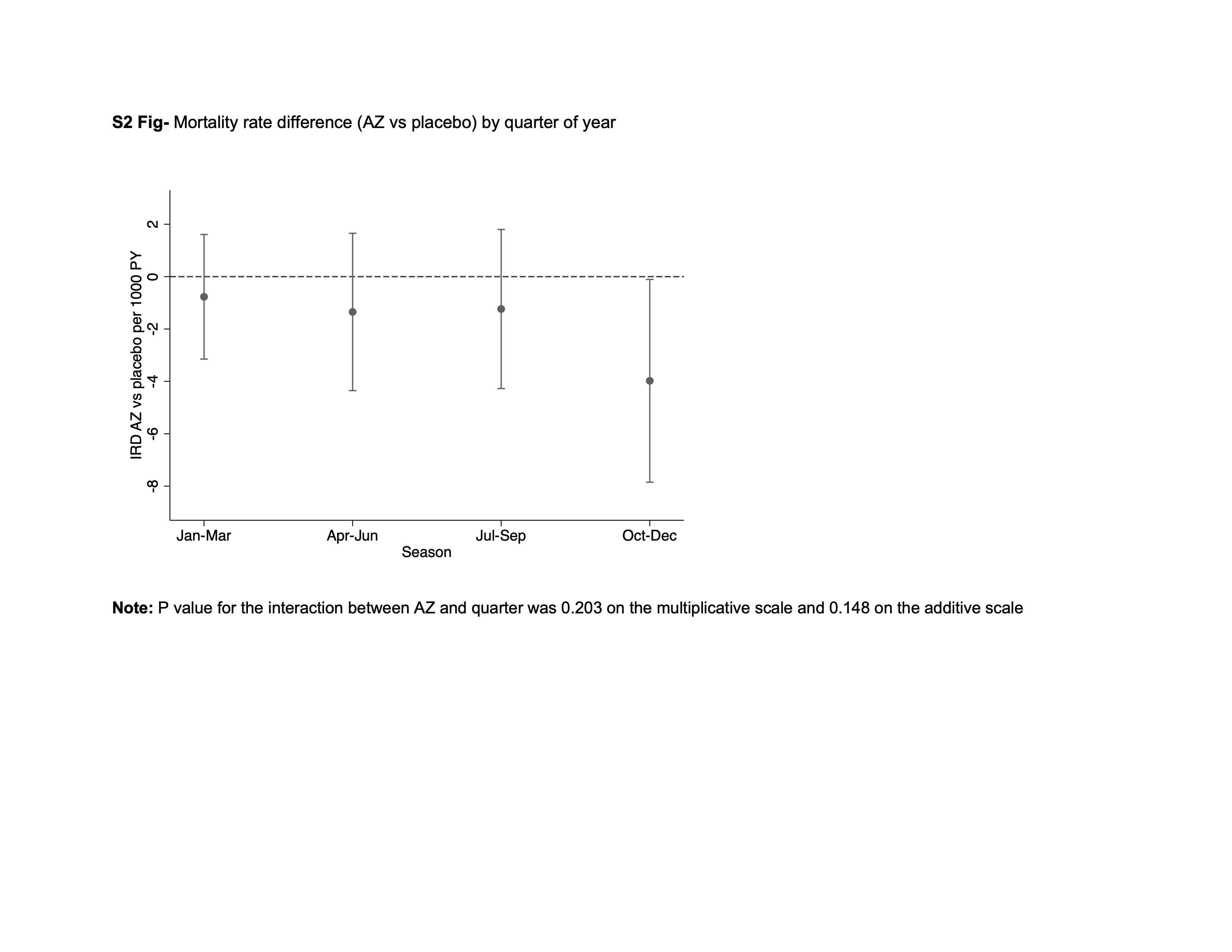

Supplement: S2 Fig — (TIFF) [file pgph.0004653.s003.tiff]

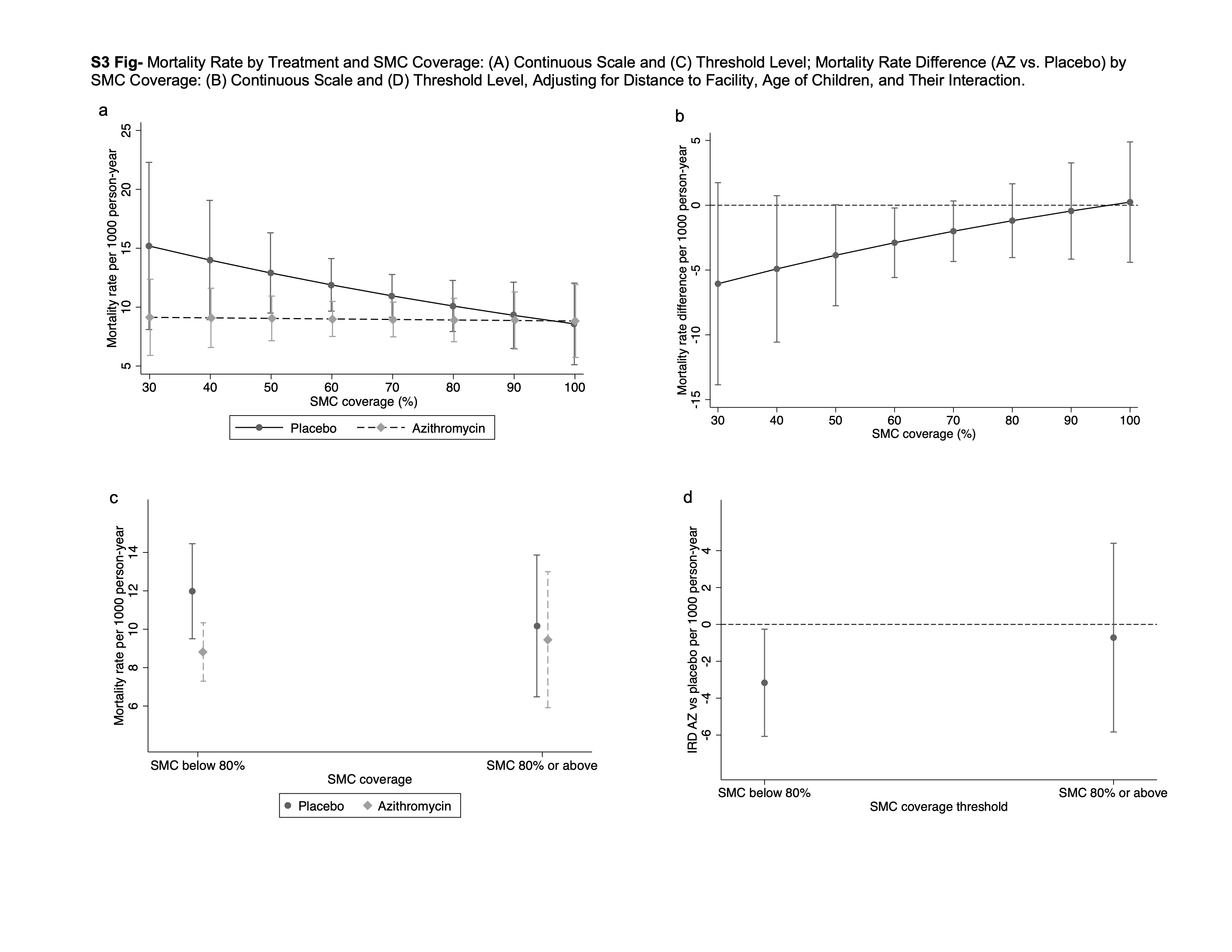

Supplement: S3 Fig — (TIFF) [file pgph.0004653.s004.tiff]

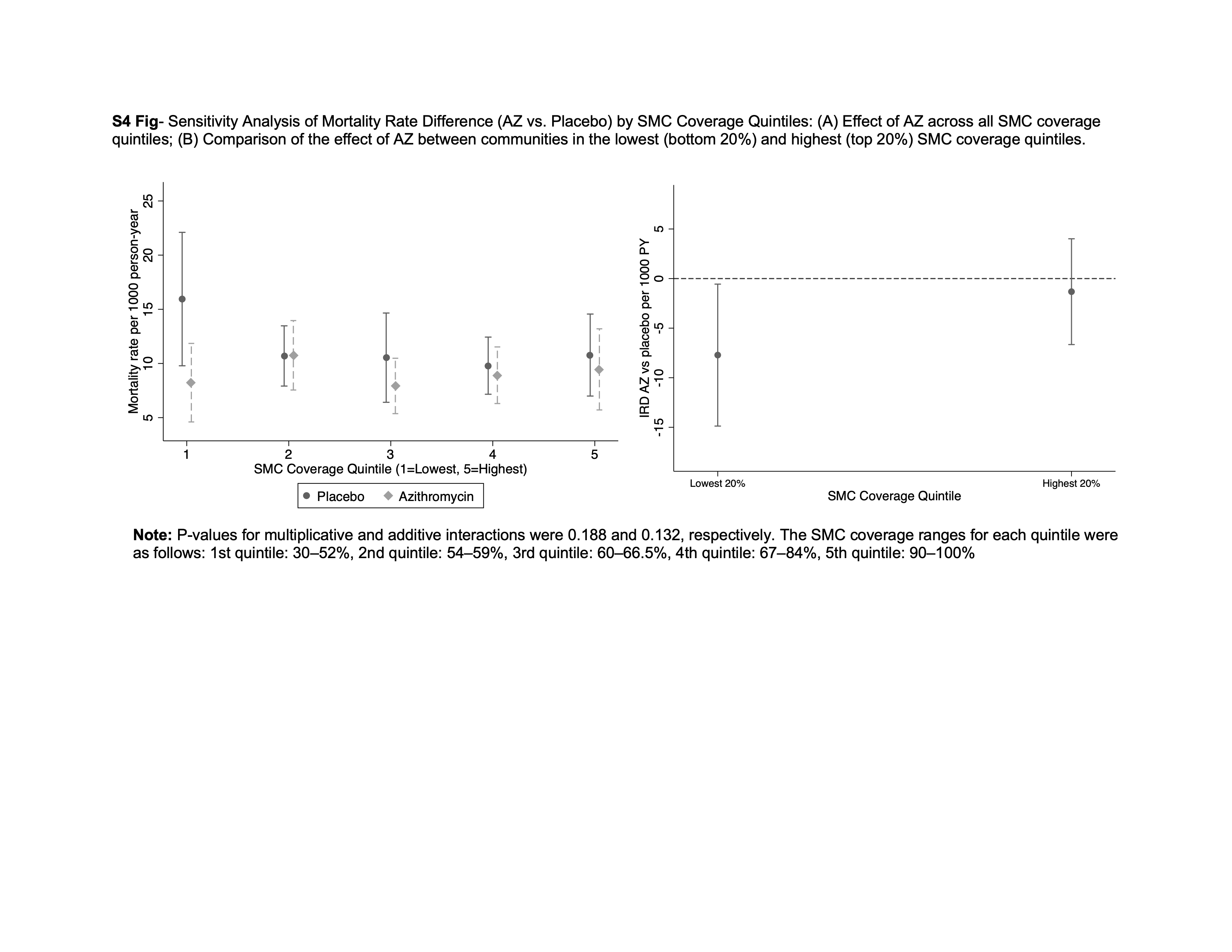

Supplement: S4 Fig — (TIFF) [file pgph.0004653.s005.tiff]
