# Supplementary material for: The role of seasonal malaria chemoprevention in the effect of azithromycin on child mortality: A secondary analysis of the CHAT cluster randomized clinical trial
Source: PLOS Glob Public Health. 2025 Sep 29;5(9):e0004653. doi: 10.1371/journal.pgph.0004653 (PMC12478956; doi:10.1371/journal.pgph.0004653)
Supplement: S1 Table — (DOCX) [file pgph.0004653.s006.docx]

| **S1 Table-** Effect of Azithromycin vs Placebo Distribution on Child Mortality by SMC coverage level | | | | | | |
| --- | --- | --- | --- | --- | --- | --- |
| **SMC coverage** | **Mortality rate per 1000 PY (all clusters)** | **Mortality rate per 1000 PY in AZ** | **Mortality rate per 1000 PY in Placebo** | **IRR (AZ vs placebo)** | **IRD (AZ vs Placebo)**  **per 1000 PY** | **Number needed to treat to prevent one death** |
| Overall | 10.3 (9.1 to 11.6) | 9.0 (7.5 to 10.4) | 11.6 (9.6 to 13.6) | 0.77 (0.6 to 0.98) | -2.6 (-5.0 to -0.1) | 387 |
| 30% | 11.6 (7.6 to 15.7) | 8.5 (4.8 to 12.3) | 14.9 (7.6 to 22.2) | 0.57 (0.2 to 0.95) | -6.4 (-14.6 to 1.8) | 157 |
| 40% | 11.2 (8.2 to 14.2) | 8.6 (5.7 to 11.6) | 13.9 (8.6 to 19.2) | 0.62 (0.3 to 0.94) | -5.2 (-11.3 to 0.8) | 191 |
| 50% | 10.8 (8.7 to 12.9) | 8.8 (6.6 to 11) | 12.9 (9.3 to 16.5) | 0.68 (0.42 to 0.93) | -4.2 (-8.4 to 0) | 240 |
| 60% | 10.4 (9 to 11.8) | 8.9 (7.3 to 10.5) | 12 (9.7 to 14.4) | 0.74 (0.54 to 0.93) | -3.2 (-6.0 to -0.3) | 318 |
| 70% | 10.1 (8.9 to 11.3) | 9 (7.5 to 10.5) | 11.2 (9.4 to 13) | 0.80 (0.62 to 0.99) | -2.2 (-4.5 to 0.2) | 456 |
| 80% | 9.8 (8.3 to 11.2) | 9.2 (7.2 to 11.1) | 10.5 (8.3 to 12.6) | 0.88 (0.62 to 1.14) | -1.3 (-4.2 to 1.6) | 775 |
| 90% | 9.5 (7.5 to 11.5) | 9.3 (6.5 to 12.1) | 9.7 (6.9 to 12.6) | 0.95 (0.56 to 1.35) | -0.4 (-4.4 to 3.5) | 2274 |
| 100% | 9.3 (6.7 to 11.8) | 9.4 (5.7 to 13.2) | 9.1 (5.5 to 12.6) | 1.04 (0.46 to 1.62) | 0.4 (-4.8 to 5.5) | 2743 |
| Interaction Coeff multiplicative scale 1.1 (0.9 to 1.3), P=0.297 | | | | | | |
| Interaction Coeff additive scale 0.08 (-0.06 to 0.21), P= 0.264 | | | | | | |
