# Supplementary material for: The role of seasonal malaria chemoprevention in the effect of azithromycin on child mortality: A secondary analysis of the CHAT cluster randomized clinical trial
Source: PLOS Glob Public Health. 2025 Sep 29;5(9):e0004653. doi: 10.1371/journal.pgph.0004653 (PMC12478956; doi:10.1371/journal.pgph.0004653)
Supplement: S2 Table — (DOCX) [file pgph.0004653.s007.docx]

| **S2 Table-** Effect of Azithromycin vs Placebo Distribution on Child Mortality by SMC coverage threshold of 80% | | | |
| --- | --- | --- | --- |
|  | **Across all clusters (N=285)** | **Coverage below 80% (N=222)** | **Coverage 80% or above (N=86)** |
| Mortality rate per 1000 PY (all clusters) | 10.3 (9 to 11.5) | 10.5 (9 to 11.9) | 9.2 (6.8 to 11.7) |
| Mortality rate per 1000 PY in AZ | 9.0 (7.5 to 10.4) | 8.9 (7.3 to 10.5) | 9.2 (5.9 to 12.6) |
| Mortality rate per 1000 PY in Placebo | 11.5 (9.4 to 13.6) | 12.1 (9.6 to 14.7) | 9.2 (5.8 to 12.7) |
| IRR (AZ vs placebo) | 0.78 (0.61 to 0.99) | 0.73 (0.56 to 0.96) | 1.0 (0.59 to 1.69) |
| IRD (AZ vs placebo) | -2.49 (-5.03 to 0.05) | -3.3 (-6.2 to -0.3) | 0.01 (-4.8 to 4.9) |
| Number Needed to treat to prevent one death | 402 | 308 | 119,306 |
| Interaction Coeff multiplicative | 1.37 (0.76 to 2.48), P= 0.301 | |  |
| Interaction Coeff additive | 0.27 (-0.19 to 0.72), P=0.247 | |  |

**Note:** Some clusters may appear in both high and low coverage counts if their estimates were above the threshold in one year and below the threshold in another year.
