# Supplementary material for: The role of seasonal malaria chemoprevention in the effect of azithromycin on child mortality: A secondary analysis of the CHAT cluster randomized clinical trial
Source: PLOS Glob Public Health. 2025 Sep 29;5(9):e0004653. doi: 10.1371/journal.pgph.0004653 (PMC12478956; doi:10.1371/journal.pgph.0004653)
Supplement: S3 Table — (DOCX) [file pgph.0004653.s008.docx]

**S3 Table**- Effect of Azithromycin vs. Placebo on Child Mortality by Season, Adjusting for SMC Coverage

|  | **Non-SMC (Jan-Jun)** | **SMC (Jul-Dec)** |
| --- | --- | --- |
| Mortality rate per 1000 PY (all clusters) | 7.9 (6.9 to 9.0) | 10.3 (9 to 11.6) |
| Mortality rate per 1000 PY in AZ | 7.5 (6.1 to 8.8) | 9.0 (7.5 to 10.4) |
| Mortality rate per 1000 PY in Placebo | 8.4 (6.8 to 10.1) | 11.7 (9.5 to 13.9) |
| IRR (AZ vs placebo) | 0.89 (0.68 to 1.15) | 0.77 (0.6 to 0.98) |
| IRD (AZ vs placebo) | -0.97 (-3.07 to 1.14) | -2.7 (-5.3 to -0.07) |
| Number Needed to treat to avert one death | 1037 | 371 |
| Interaction Coeff multiplicative scale | 0.87 (0.65 to 1.16), p= 0.335 | |
| Interaction Coeff additive scale | -0.21 (-0.57 to 0.16), p=0.268 | |

**Note:** Table shows results from sensitivity analyses adjusting for coverage
