# Supplementary material for: The role of seasonal malaria chemoprevention in the effect of azithromycin on child mortality: A secondary analysis of the CHAT cluster randomized clinical trial
Source: PLOS Glob Public Health. 2025 Sep 29;5(9):e0004653. doi: 10.1371/journal.pgph.0004653 (PMC12478956; doi:10.1371/journal.pgph.0004653)
Supplement: Attachment — Submitted filename: Response to Reviewers_PLOS Global Public Health_Aug16.pdf [file pgph.0004653.s010.pdf]

August 16, 2025

Editor: Alejandro Torrado Pacheco, Ph.D.

PLOS Global Public Health

RE: MANUSCRIPT ENTITLED, "The role of Seasonal Malaria Chemoprevention in the effect of Azithromycin on Child Mortality: A Secondary Analysis of the CHAT Cluster Randomized Clinical Trial"

Dear Dr. Alejandro Torrado Pacheco,

We are grateful to have the opportunity to respond to the Reviewers' and Editor's comments and queries. We truly appreciate their time and effort in assessing our manuscript. Our responses follow below:

Journal Requirements/edits:

1. Please provide additional details regarding participant consent. In the ethics statement in the Methods and online submission information, please ensure that you have specified (1) whether consent was informed and (2) what type you obtained (for instance, written or verbal, and if verbal, how it was documented and witnessed). If your study included minors, state whether you obtained consent from parents or guardians. If the need for consent was waived by the ethics committee, please include this information.

Response: The secondary analysis was conducted using data collected under the original CHAT trial consent and IRB approval. Written informed consent was obtained from the caregiver of each participant at the time of enrollment in the CHAT study. We have added in the ethics statement under methods that "Written informed consent was obtained from the caregiver of each participant."

Reviewers' comments:

Comments to the Author

Reviewer #1: This was a post hoc secondary analysis of the CHAT trial and additionally included SMC data from NMCP. The primary outcome for these analyses was all-cause mortality, assessed using a biannual census. The sample size was based on childhood mortality for the overall AZ versus placebo comparison in the CHAT trial. The basic design and analysis plan had been previously reviewed and accepted as valid.

The sensitivity analysis in this renewed or revisited setting accounting for possible appropriate covariates or confounders adjusted for SMC coverage in the analysis by SMC season and they categorized the year into quarters, to evaluate during which time of the year AZ distribution yielded the greatest benefit against mortality. Lastly, they examined the role of SMC coverage in the effect of AZ on child mortality adjusting for age of children and distance to health facility, and their interactions with AZ as both may influence the level of SMC coverage and affect the benefit of AZ for mortality. Apparently in the Results section the sensitivity results did not cause concern.

For appropriate coverage analyses they used Poisson regression models with robust standard errors and similar methods for examining interactions on additive and multiplicate scales. Coverage and mortality investigations are seen in the tables and figures in the text and supplementary materials.

Despite the lack of statistical significance on several fronts, the analysis was done appropriately and the descriptive summary in the discussion was comprehensive giving several limitations, one of which perhaps was the sample size limitation resulting in what the authors describe as lack of power which can happen in a secondary analysis setting.

Response: Thank you very much for your thoughtful and generous review. We sincerely appreciate your careful reading of the manuscript and your recognition of the design, analytical approach, and limitations of this secondary analysis. Your supportive comments are encouraging and greatly valued. Thank you again for your helpful feedback.

Reviewer #2: The authors have utilized Poisson regression with person-time at risk as an offset (likely using log(person-time)) to estimate incidence rates and incidence rate ratios. While Poisson regression is appropriate for count-based outcomes, its direct application to mortality data aggregated across time and clusters can lead to overly simplistic interpretations due to assumptions of a constant hazard rate over the observation period. Specifically:

1. Constant Hazard Assumption
The Poisson regression model used by authors implicitly assumes a constant hazard rate (mortality risk) over the duration of follow-up periods. This assumption is questionable given the biologically plausible temporal variability in mortality rates, particularly related to the seasonal effects of malaria and other infectious diseases. Such constant-hazard assumptions typically underestimate effects (particularly if hazards decline or vary significantly over time), potentially reducing power and precision.

Recommendation:
Consider employing survival analysis methods such as the Cox proportional hazards

model (multiplicative effects) or other flexible models (e.g., accelerated failure-time models, piecewise exponential additive mixed models). These models naturally accommodate variable hazards over time and can explicitly handle right-censoring and variable follow-up periods inherent in longitudinal cohort data. Cox models, especially with robust standard errors or frailty terms (random effects to account for clustering at the community level), would more appropriately reflect the underlying data structure and temporal variations. The authors' categorization of SMC administration as time periods (January–June vs July–December) could be treated as a time-varying covariate to dynamically model how its effect on mortality changes throughout the study period. Survival models may also show an improved power when using SMC coverage as a continuous covariate.

Response: Thank you for this thoughtful and detailed suggestion. We agree that modeling time-varying hazards is important when working with individual-level survival data with precise timing of events. However, in our study, the primary analysis was conducted at the cluster-phase level, with counts of deaths and person-time aggregated over 6-month intervals/phases, reflecting the design of the original CHAT trial. Both exposure (treatment assignment) and SMC coverage were defined at the community level, and mortality was assessed through biannual censuses, with no exact dates of death/events for most children. As such, individual-level survival analysis methods like Cox proportional hazards models may not be applicable to the structure of our data.

We selected Poisson regression (with a log(person-time) offset and robust standard errors), which is typically used in prior similar studies and cluster-randomized trials assessing mortality rates at the community level (Keenan, 2018, PMCID: PMC5849140; O'Brien, 2024, PMID: 39167806; Chandramohan 2019 PMID: 30699301). This approach aligns with the statistical analysis plan used in the original CHAT trial (Oldenburg 2024, PMCID: PMC10865159) and maintains consistency with the design and level of inference for our study.

Our primary objective remains to assess whether seasonal variation and community level SMC coverage modifies the effect of a community-level AZ intervention on population-level mortality rates. A shift to individual-level survival modeling would not enhance the validity of our findings and may, in fact, introduce bias by attempting to model hazard functions without precise event times.

For these reasons, we believe Poisson regression remains the most appropriate analytic approach for our data and research question although we appreciate the suggestion.

2. Additive versus Multiplicative Interactions

The authors reported both additive and multiplicative interactions. While reporting both provides thoroughness, it may introduce interpretive complexity. A clearer rationale for choosing additive or multiplicative interactions is beneficial.

Recommendation: Provide explicit epidemiological justification or interpretation for choosing to report interactions on both scales. Consider emphasizing the scale most relevant to public health implications clearly and succinctly in the discussion.

Response:

Thank you for the recommendation.

We evaluated interaction on both the additive and multiplicative scales to provide a comprehensive assessment of effect modification as studies recommend (Knol and VanderWeele 2012 PMCID: PMC3324457).

The additive scale indicates whether the effect of the intervention is greater in one subpopulation or season than in another, making it useful for targeting specific populations or timing and for resource allocation by identifying subgroups with the greatest absolute benefit. In contrast, multiplicative interactions assess relative differences in effect and provide insight into differences across risk groups on a relative scale (Knol and VanderWeele 2012 PMCID: PMC3324457).

Evaluating both scales allows for a richer understanding of how the effect of the intervention varies across seasonal contexts and subpopulations as interactions may sometimes be present on one scale but not in another.

As suggested, we have added our rationale for evaluating interaction on both scales in the statistical analysis section.

3. Sensitivity Analysis: Dichotomizing SMC Coverage
The authors categorized coverage at an 80% threshold. While using established thresholds is informative, the sensitivity analysis might gain clarity and impact from a more pronounced contrast.

Recommendation:

Perform an additional sensitivity analysis comparing communities in the highest quintile of coverage (top 20%) with the lowest quintile (bottom 20%), providing a stark and potentially more insightful contrast of extremes in SMC coverage.

Response: As suggested, we have added a supplementary figure assessing differences in the effect of AZ by SMC coverage quintiles. We present the effect across all quintiles, as well as a comparison between the highest and lowest quintiles. Although the interaction did not reach statistical significance (as with the other analyses) it shows a consistent pattern suggesting that the effect of AZ may have been slightly greater in

communities with lower SMC coverage. Supplementary Figure 4 displays the results of these sensitivity analyses. This sensitivity analysis is also now added in the methods and results.

4. Visual Presentation of Results
Some figures largely duplicates information presented in Tables (eg Fig 5 and Table 3 - Fig 4 and Table 2 - Fig 6 and Table 4).

Response: Thank you for this suggestion. We reviewed the overlap between tables and figures and have streamlined the presentation to reduce redundancy. Specifically, Tables 3 and 4 have been moved to the supplementary materials (now Supplementary Tables 1 and 2), as their corresponding Figures 5 and 6 convey the key findings visually. However, we have retained Table 2 alongside Figure 4 to provide at least one detailed display of key estimates including mortality rates, rate ratios, rate differences, number needed to treat, and p-values for readers who may wish to reference the full numerical results.

5. Type I error control
The authors investigated many different interactions with SMC coverage (through time periods, as a continuous measure and also as a dichotomous aggregated variable) with treatment. Looking at both incidence rate differences and incidence rate ratios. While this can look like a thorough exploratory investigation, if one of the methods would yield a significant result, so SMC coverage <80% shows a significant AZ vs Placebo IRD and IRR, with the many tests and comparisons that were performed, could the likelihood of a type I error here be higher?

Response: Thank you for this point regarding the potential for inflated Type I error due to multiple comparisons. In our analysis, we focused on three primary interaction tests: (1) SMC coverage as a continuous variable, (2) SMC coverage using a binary threshold (<80% vs ≥80%), and (3) season of year. These were based on prior hypotheses defined before conducting additional exploratory sensitivity analyses.

As suggested, we considered the implications of multiple testing and how these would appear when applying a Bonferroni correction for these three primary interaction models. This would set a conservative significance threshold of $p < 0.017$ (0.05 divided by 3). None of our interaction effects meet this threshold, and in fact, all p-values exceeded even the conventional 0.05 threshold.

Given the relatively limited number of primary interaction tests (aside from the sensitivity analyses conducted to assess the robustness of results) and the already limited power of this secondary analysis, applying further correction to prevent false positives may be unnecessary. We have taken care not to overstate any findings, emphasizing

consistency and directionality rather than isolated p-values, and have clearly stated that they did not reach statistical significance.

We appreciate the reviewer's insight and have added the following note in the discussion to reflect this consideration.

"Although no results reached statistical significance, the potential for Type I error remains due to multiple comparisons inherent in exploratory analyses."

On behalf of all authors, we wish to reiterate our gratitude and appreciation for being given the opportunity to respond to the Reviewers' comments as well as for being considered for publication. If there are any further questions or comments, please do not hesitate to contact me.

Sincerely,

Elisabeth Gebreegziabher, PhD
